# Supplementary material for: A Novel Antimicrobial Peptide Sp-LECin with Broad-Spectrum Antimicrobial Activity and Anti-Pseudomonas aeruginosa Infection in Zebrafish
Source: Int J Mol Sci. 2022 Dec 23;24(1):267. doi: 10.3390/ijms24010267 (PMC9820466; doi:10.3390/ijms24010267)
Supplement: Supplementary file 1 [file ijms-24-00267-s001.zip › Supplementary Materials.pdf]

Supplementary Materials

For

A novel antimicrobial peptide Sp-LECin with broad-spectrum antimicrobial activity and anti-*Pseudomonas aeruginosa* infection in zebrafish

Yan-Chao Chen [1,†], Wanlei Qiu [1,†], Weibin Zhang [1], Jingrong Zhang [1], Roushi Chen [1], Fangyi Chen [1,2,3,*] and Ke-Jian Wang [1,2,3,*]

[1] State Key Laboratory of Marine Environmental Science, College of Ocean & Earth Sciences, Xiamen University, Xiamen 361102, China

[2] State-Province Joint Engineering Laboratory of Marine Bioproducts and Technology, College of Ocean & Earth Sciences, Xiamen University, Xiamen 361102, China

[3] Fujian Innovation Research Institute for Marine Biological Antimicrobial Peptide Industrial Technology, College of Ocean & Earth Sciences, Xiamen University, Xiamen 361102, China

* Correspondence: chenfangyi@xmu.edu.cn (F.C); wkjian@xmu.edu.cn (K.-J.W)

† These authors made equal contributions

A

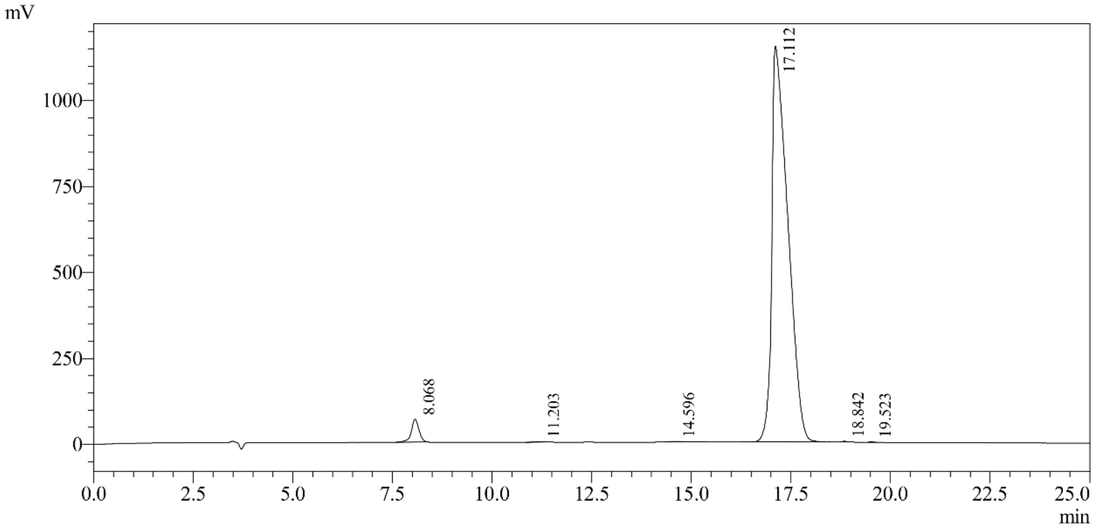

Peak Table

Detector A Channel 1 220nm

Peak#	Ret. Time	Area	Height	Area %
1	8.068	886949	66647	2.676
2	11.203	24653	1352	0.074
3	14.596	26581	1529	0.080
4	17.112	32181161	1150181	97.083
5	18.842	20519	2674	0.062
6	19.523	8062	1402	0.024
Total		33147924	1223785	100.000

B

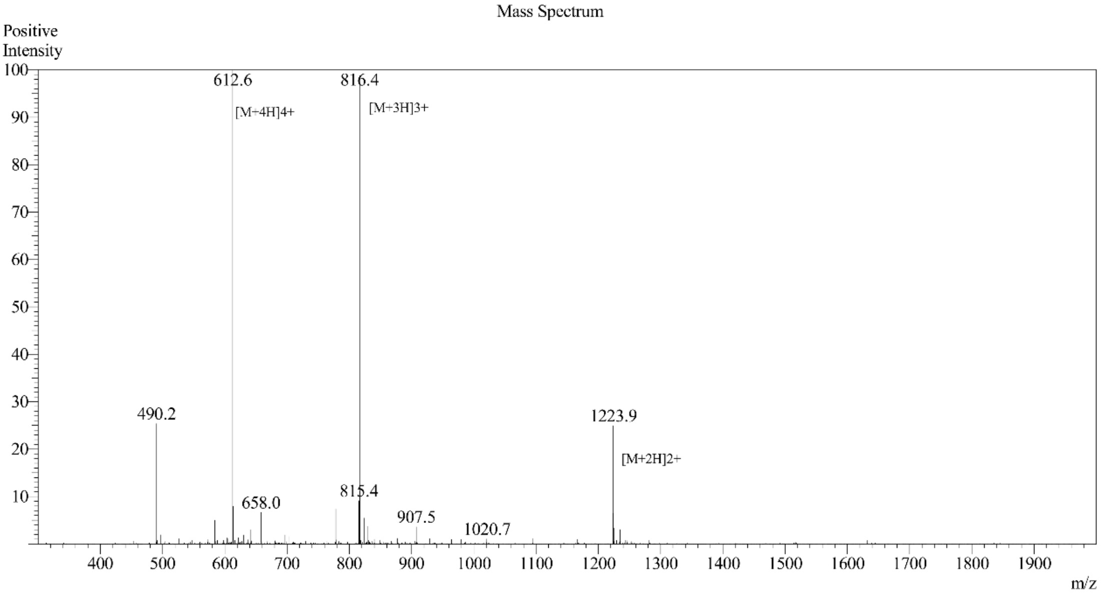

Sample Information
Month-Day Processed : 02/21/22
Time Processed : 10:26:23 AM
Injection Volume : 0.4
Sample Name : Sp-LECin
Sample ID : C938THA170-1
Theoretical MW : 2445.98
Observed MW : 2446.4

Interface :ESI
Nebulizing Gas Flow :1.5L/min
CDL Temp :250
Block Temp :200

Equipment : GK11010007
Interface Bias : +4.5 kV
Drying Gas Flow :5 L/min
T.Flow :0.2 ml/min
B.conc :50%H2O/50%MeOH

Figure S1. The HPLC (A) and MS (B) data of Sp-LECin.
